# Are Thoracic Aortic Aneurysm Patients at Increased Risk for Cardiovascular Diseases?

**DOI:** 10.3390/jcm12010272

**Published:** 2022-12-29

**Authors:** Onur B. Dolmaci, Sulayman El Mathari, Antoine H. G. Driessen, Robert J. M. Klautz, Robert E. Poelmann, Jan H. N. Lindeman, Nimrat Grewal

**Affiliations:** 1 Department of Cardiothoracic Surgery, Leiden University Medical Center (LUMC), 2333 ZA Leiden, The Netherlands; 2 Department of Cardiothoracic Surgery, Amsterdam University Medical Center, 1105 AZ Amsterdam, The Netherlands; 3 Institute of Biology, Animal Sciences and Health, Leiden University, 2333 ZA Leiden, The Netherlands; 4 Department of Vascular Surgery, Leiden University Medical Center (LUMC), 2333 ZA Leiden, The Netherlands; 5 Department of Anatomy and Embryology, Leiden University Medical Center, 2333 ZA Leiden, The Netherlands

**Keywords:** aortic dilatation, thoracic aortic aneurysm, abdominal aortic aneurysms, bicuspid aortic valve, coronary artery disease, cardiovascular risk management

## Abstract

Objectives: Abdominal aortic aneurysms are associated with a sharply increased cardiovascular risk. Cardiovascular risk management is therefore recommended in prevailing guidelines for abdominal aneurysm patients. It has been hypothesized that associated risk relates to loss of aortic compliance. If this hypothesis is correct, observations for abdominal aneurysms would also apply to thoracic aortic aneurysms. The objective of this study is to test whether thoracic aneurysms are also associated with an increased cardiovascular risk burden. Methods: Patients who underwent aortic valve or root surgery were included in the study (*n* = 239). Cardiovascular risk factors were studied and atherosclerosis was scored based on the preoperative coronary angiographies. Multivariate analyses were performed, controlling for cardiovascular risk factors and aortic valve morphology. Comparisons were made with the age- and gender-matched general population and non-aneurysm patients as control groups. A thoracic aortic aneurysm was defined as an aortic aneurysm of ≥45 mm. Results: Thoracic aortic aneurysm was not associated with an increased coronary atherosclerotic burden (*p* = 0.548). Comparison with the general population revealed a significantly higher prevalence of hypertension (61.4% vs. 32.2%, *p* < 0.001) and a lower prevalence of diabetes (1.4% vs. 13.1%, *p* = 0.001) in the thoracic aneurysm group. Conclusions: The extreme cardiovascular risk associated with abdominal aortic aneurysms is location-specific and not explained by loss of aortic compliance. Thoracic aortic aneurysm, in contrast to abdominal, is not part of the atherosclerotic disease spectrum and, therefore, cardiovascular risk management does not need to be implemented in treatment guidelines of isolated thoracic aneurysms. Hypertension should be treated.

## 1. Introduction

While the primary concern in abdominal aortic aneurysm (AAA) disease is rupture, AAA is also associated with a sharply increased cardiovascular risk that by far exceeds the risk for patients with a previous myocardial infarction, cerebrovascular or peripheral artery disease, and that is similar to that of patients with poly-vascular disease [1,2]. AAA patients are therefore considered at extremely high-risk in the current cardiovascular risk prevention guidelines [3].

Recommendations with respect to cardiovascular risk prevention are currently not included in the treatment guidelines for patients with a thoracic aortic aneurysm (TAA) [4]. A critical question is whether the recommendations with respect to risk management for AAA should be extended to TAA. It has been hypothesized that the increased cardiovascular risk in AAA patients is secondary to changes in aortic wall compliance (and thus an increased cardiac afterload), and consequently that an increased cardiovascular risk is also observed in patients with a TAA. An alternative but nonexclusive explanation is that the increased cardiovascular risk for AAA patients is specific for AAA, and for example reflects the fact that AAA disease is part of the atherosclerotic spectrum of diseases. In the latter scenario, the observed increased cardiovascular risk will be specific, or more prominent for aneurysms of the abdominal aorta.

To address this question, and to test whether recommendations with respect to cardiovascular risk management for AAA patients should be extended to TAA patients, an evaluation of the cardiovascular disease burden in patients with a TAA was considered relevant. In order to avoid interference by inclusion of patients with bicuspid aortic valve (BAV) disease, a predilection factor for thoracic aortic aneurysms [5,6] and a possible negative risk factor for atherosclerosis, sensitivity analyses were performed for TAA associated with either a tricuspid (TAV) or a bicuspid aortic valve [7,8].

## 2. Materials and Methods

This retrospective study was conducted at the Leiden University Medical Center (LUMC) in the Netherlands. Approval for this study was granted by the medical ethics committee of the Leiden University Medical Center, and the need for patient consent was waived. The study includes on all consecutive 239 patients who underwent an aortic valve or root and/or ascending aortic surgery due to an underlying aortic (root) aneurysm and/or aortic regurgitation between January 2006 and January 2020. Cardiovascular risk factors and coronary atherosclerotic disease burden were extensively mapped. The patients were divided into two groups: patients with thoracic aortic aneurysm (TAA) and the non-aneurysmal control group (non-TAA). Thoracic aorta aneurysms were defined as an ascending aorta diameter of ≥45 mm. Subgroup analyses were performed for patients with a bicuspid and tricuspid aortic valve. BAV and TAV were defined and classified according to the Sievers classification based on the intraoperative observation of the surgeon.

Transcatheter procedures, patients under the age of 18, patients with active endocarditis, aortic dissection, previous aortic valve surgery and/or no preoperative coronary angiogram were excluded.

The cardiovascular risk profiles and atherosclerotic disease burden were compared with an age- and gender-matched general population (available through the Dutch general practitioners’ NIVEL Primary Care Database (NPCD, 2019)) [8,9]. The NPCD is a longitudinal database in which data from Dutch general practitioners are collected for study purposes. The database provides a representative sample of the Dutch population [9]. Included diagnoses are coded by the primary care physicians using the International Classification of Primary Care (ICPC) [9]. Codes used in this study were: K74, K75, K76, T90.1, T90.2, K86, K87, T93.01, T93.03 and T93.04.

### 2.1. Study Parameters

The electronic health records were systematically searched to obtain data regarding demographics, coronary artery disease (CAD) history and CAD risk factors (a family history of CAD (aged younger than 65)), hypertension (defined as a blood pressure of ≥160/95 mm Hg or the use of antihypertensive drugs), diabetes mellitus (defined as either a blood glucose level of ≥7.0 mmol/L on two separate (fasted) occasions, a glucose level of ≥11.1 mmol/L plus symptoms of hyperglycemia, and/or use of anti-diabetic medication), tobacco usage and the body mass index) [10]. Most aortic dimensions were obtained from preoperative computed tomographies. Transthoracic ultrasound estimates were used in cases in which a computed tomography was not performed. The type of surgery, concomitant procedures and the aortic valve morphology (including the Sievers classification for BAV patients) were obtained from the surgical reports.

CAD risk factors for the general population (hypertension, diabetes mellitus and hypercholesterolemia) were obtained from the NPCD database. In the NIVEL primary care database hypertension is conservatively defined as a blood pressure ≥ 160/95 mm Hg instead of the prevailing guidelines definition of a blood pressure of ≥140/80 mm Hg. In order to allow cross-group comparison, hypertension was defined as a systolic pressure of ≥160 mm Hg and/ or a diastolic pressure of ≥95 mm Hg on two separate occasions, and/or as the usage of antihypertensive drugs [9] for the whole study population. Hypercholesterolemia was defined as a total cholesterol level of ≥6.5 mmol/L or the used lipid-lowering medications [9]. Diabetes was defined as described above. A history of coronary artery disease was defined as a previous myocardial infarction or unstable angina pectoris.

### 2.2. Coronary Sclerosis

**Figure 1:**
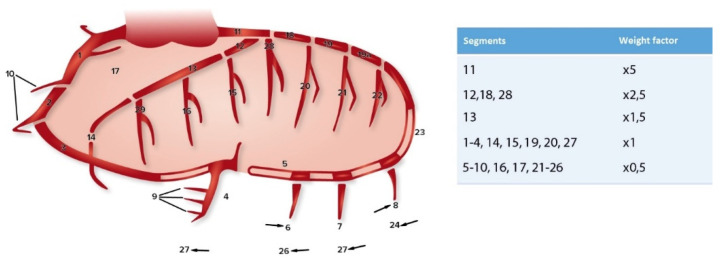
Coronary artery segments (according to CASS) and the corresponding weight factors used for the CAGE score [11,13].

Preoperative coronary angiographies (up to one year before surgery) were used to score the severity of coronary sclerosis for each patient. The coronary artery sclerosis greater than or equal to 20 and 50 (CAGE ≥ 20 and CAGE ≥ 50) method was used to score the extent and severity of the coronary artery sclerosis [11,12,13]. This method scores non-obstructive coronary sclerosis (=CAGE 20, 20–49% coronary obstruction) and obstructive coronary sclerosis (=CAGE 50, ≥50% coronary obstruction) for 28 different coronary segments (Figure 1). The coronary angiographies were independently scored by two researchers.

As angiographies performed in the general population were not available for this study, CAD could not be compared between the general population and the study groups.

### 2.3. Statistical Analysis

Normally distributed continuous variables are presented as mean ± standard deviation (SD), while non-normal distributed continuous variables are presented as median and interquartile range (IQR). Categorical data are presented as frequencies and percentages. Skewness, kurtosis and normality tests were performed for all variables. *t*-tests were performed to analyze continuous variables and a log transformation was performed when needed. Categorical data were analyzed using Fischer’s exact test. A linear regression was performed to model the relationship of two continuous variables. Multivariate regression analyses were performed after initially performing univariate analyses (including all variables with *p* < 0.2) on the whole group to model the dependence of an ascending aortic aneurysm and the aortic valve morphology on the CAGE ≥ 20 and CAGE ≥ 50 scores, controlling for CAD risk factors (e.g., age at surgery, gender, body mass index, the smoking status, hypertension, hypercholesterolemia, diabetes mellitus, previous myocardial infarction or angina pectoris, a family history of CAD and aortic valve morphology). A *p* value of <0.05 was considered to be significant. All statistical analyses were conducted using IBM SPSS for Windows version 25.0.

## 3. Results

### 3.1. Baseline Characteristics Study Populations

This study includes 70 patients with a thoracic aortic aneurysm (TAA, ≥45 mm) with a median age of 64 years (74% males) (Table 1). The study incorporates two control groups: an age- and sex-matched cohort from the general population (Supplementary Table S1), and a second control group of 169 patients who required aortic valve or root replacement in the absence of a dilated ascending aorta (non-TAA, <45 mm) with a median age of 62 years (73% males) (Table 1). Procedural findings of the two groups that underwent root replacement are provided in Table 2.

### 3.2. Cardiovascular Risk profiles

Compared to the age- and sex-matched general population (Supplementary Table S1), patients with a TAA presented with a higher prevalence of hypertension (61.4% (cases) vs. 32.2% (general population), *p* < 0.001). The prevalence of hypercholesterolemia was similar in the TAA group and the general population (*p* = 0.524), whereas diabetes mellitus was less prevalent in the TAA group as compared to the general population (1.4% vs. 13.1%, *p* = 0.001). A history of CAD was equally common in TAA patients and the general population (*p* > 0.197).

Comparison of cardiovascular risk profiles of TAA and non-TAA cohorts indicated a similar prevalence of hypertension and hypercholesterolemia. Diabetes mellitus was less prevalent in the TAA group compared to the non-TAA group (1.4% vs. 9.5%, *p* = 0.027). A history of CAD was equally common in TAA and non-TAA patients (*p* > 0.05).

**Figure 2:**
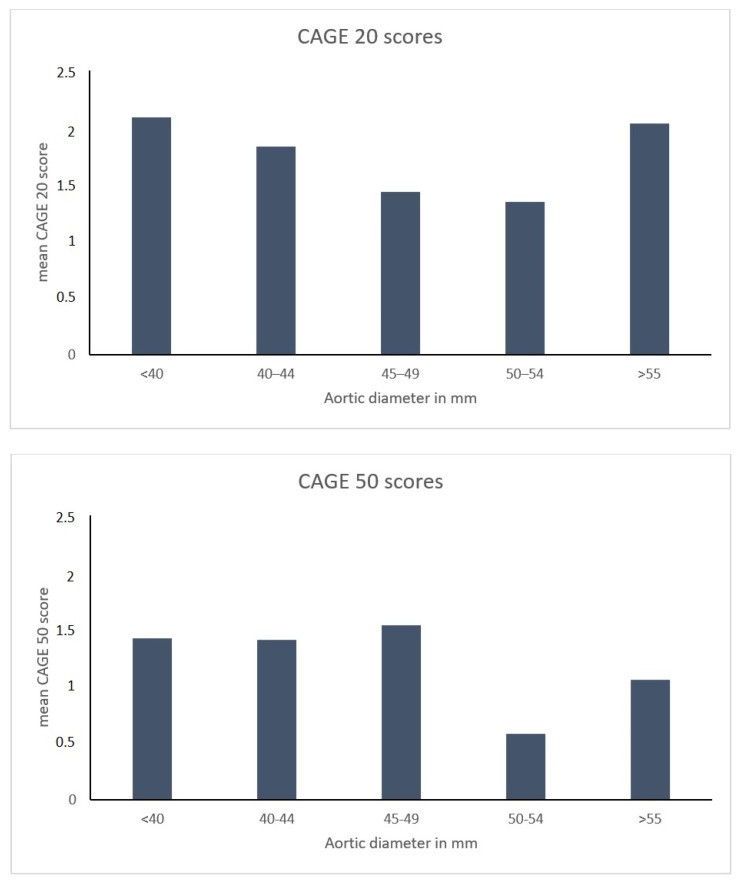
CAGE scores per diameter. Sensitivity analysis using ascending aortic diameter as a continuous variable did not indicate a correlation between CAGE 20 scores (upper figure, *p* = 0.894) or CAGE 50 scores (lower figure, *p* = 0.317) and aortic diameter.

Availability of coronary angiographies and per-operative findings allowed for a further and more in-depth comparison of the coronary atherosclerosis burden in the TAA patients and non-TAA controls. CAGE 20 and 50 scores in the TAA group and the non-TAA group were similar (1.65 SD 2.4 and 0.98 SD 2.4, vs. 2.03 SD 2.5 and 1.42 SD 2.9, respectively (AUC = 0.46, *p* = 0.259 and AUC = 0.46, *p* = 0.548)). Findings were not influenced by correction for CAD risk factors (hypertension, diabetes mellitus and/or hypercholesterolemia) in a multivariate analysis. A sensitivity analysis using ascending aortic diameter as a continuous variable did not indicate a correlation between CAGE 20 scores (*p* = 0.894) or CAGE 50 scores (*p* = 0.317) and aortic diameter (Figure 2).

The incidence of concomitant coronary artery bypass procedures with the root replacement was similar in the non- and TAA groups (15.7% resp. 20.7%, *p* = 0.471).

### 3.3. Aortic Valve Morphology and Coronary Artery Disease Burden

Approximately one third (36%) of the patients requiring aortic root replacement presented with a BAV. BAV has previously been associated with a lower atherosclerosis burden [7,8], and consequently conclusions might be interfered by inclusion of BAV patients. For this reason, a sensitivity analysis comparing the cardiovascular risk profile and the atherosclerotic disease burden in BAV and TAV patients was considered relevant. The proportion of BAV patients in the TAA and non-TAA control group was similar (*p* = 0.057). Baseline and perioperative characteristics of the BAV and TAV patients are summarized in Supplementary Tables S2 and S3, respectively, and the Sievers classifications of the BAV patients is shown in Supplementary Figure S1. Since BAV patients were on average 13 years younger than TAV patients (54 vs. 67 years, *p* < 0.001) observations for BAV and TAV patients were compared against the general population means (Table 3).

Although TAV was associated with lower CAGE 20 (AUC = 0.71, OR 1.49 (95% CI 1.26–1.76); *p* < 0.001) and CAGE 50 scores (AUC = 0.59, OR 1.13 (95% CI 1.003–1.27); *p* = 0.045), the difference for CAGE 50 scores was lost following multivariate analysis correcting for body mass index, diabetes mellitus, hypercholesterolemia, previous myocardial infarction, instable angina pectoris, family history of CAD, a family history of CAD and aortic dilatation. (OR 1.07 (95% CI 0.95–1.22); *p* = 0.258). CAGE 20 scores remained lower following correction for age, hypertension, hypercholesterolemia, previous myocardial infarction, family history of CAD and a history of smoking in a multivariate analysis (see Supplementary Figure S2).

A sensitivity analysis correcting for the aortic valve morphology showed no deviation in cardiovascular risk profile between TAA and non-TAA patients as described above.

## 4. Discussion

Abdominal aortic aneurysms are associated with a high cardiovascular risk burden that equals that of poly-vascular disease [2]. This high vascular risk is held responsible for the profound residual (rupture-independent) excess mortality in these patients [1,2]. As a consequence, AAA patients are classified as extremely high-risk patients in prevailing guidelines [4].

Mechanistically, this extreme cardiovascular risk has been attributed to the increased aortic stiffness, and loss of Windkessel function [14,15,16] as result of the pathological wall remodeling in the disease [17]. If the Windkessel hypothesis is valid, the same phenomenon will also apply to, and may even be more outspoken for the more proximal thoracic aneurysms [18]. So far, associations between thoracic aortic aneurysmal disease and cardiovascular risk (and risk factors) are not clear [19,20,21,22,23], and no specific recommendations with respect to cardiovascular risk management exist for these patients. This study therefore aimed to determine whether an increased cardiovascular risk is generic for aortic aneurysms, and thus that the observations for AAA also apply to TAA.

The evaluation focusses on patients who underwent thoracic aneurysm replacement and were compared with two control groups. A surgical control group (with a non-dilated ascending aorta (<45 mm)) was included in order to reduce the impact of confounding-by-indication, and because of the level of detail of the information available (e.g., coronary angiograms). Since the interpretation of data from groups that underwent surgery might be impacted by shared common risk of causative risk factors (such as underlying atherosclerotic disease), a second, population-based reference group was considered relevant. Conclusions for both reference groups were uniform and showed comparable cardiovascular risk profiles and atherosclerosis burden with TAA patients, showing that the association between AAA and atherosclerosis is disease-specific. These findings challenge the Windkessel hypothesis, and demonstrate that the presence of an isolated TAA is not an indication for cardiovascular risk management.

The observed lower prevalence of diabetes mellitus in the TAA patients, confirms and extends the apparent paradoxical negative (protective) association between diabetic disease and AAA disease to thoracic aortic aneurysms [24,25,26,27,28]. Considering the profound biological differences in disease etiology between AAA and TAA, we hypothesize that this observation implies that the protective effects of diabetes are most likely mediated by an effect on a common factor such as mesenchymal cell and/or matrix biology and less likely through an effect on one of the effector mechanisms such as inflammation. Our results indicate that the aortic valve morphology does not interfere with this interaction.

In the light of the reported association between valve morphology and atherosclerotic disease burden [7,8], a sensitivity analysis was performed in order to test for possible contrasts between the two aortic valve types. This analysis did not indicate an association between aortic valve morphology and cardiovascular risk profiles, and/or differences in coronary sclerosis or coronary revascularization.

### Limitations

Limitations are present due to the retrospective study, single center design. Patients who were treated with a transcatheter procedure were excluded, who usually are older and sicker than surgical patients. Since the emergency surgeries of aortic dissection patients could lead to incomplete pre-operative data regarding the cardiovascular risk profile, these patients were excluded to enable more reliable comparisons. This exclusion however could contribute to inclusion bias. Differences in cardiovascular risk profiles between TAA, non-TAA, BAV and TAV patients were addressed by corrections and sensitivity analyses in order to minimize their impact, yet it cannot be excluded that the corrections were incomplete. Although aspects as confounding by indication were minimized by the inclusion of a non-TAA group, differences in medical decision making remain. Finally, the definition of hypertension (>160 mm Hg) used in this study was dictated by the definition applied in the NPCD primary care registry. The threshold is higher than the consensus threshold. It is assumed that the impact of this more conservative threshold is limited since the large majority of in-hospital patients were scored as hypertensive based on the use of antihypertensive drugs the change in definition did not make a difference. Given the extreme risk of AAA, we consider it unlikely that the negative conclusions from this study are caused by a low statistical power.

## 5. Conclusions

The extreme cardiovascular risk associated with AAA is location-specific and not explained by loss of aortic compliance. Cardiovascular risk management does not need to be implemented in the standard treatment guidelines of isolated TAA. Cardiovascular risk management, however, should be provided upon indication in individuals with an increased risk profile.

## Figures and Tables

**Table 1:** Baseline characteristics.

	TAA	Non-TAA		
Characteristic	*n* = 70	*n* = 169	OR (95% CI)	*p*-Value
Male	52 (74.3)	123 (72.8)	0.93 (0.49–1.75)	0.873
Age at surgery	64 (54–73)	62 (51–70)	1.02 (0.99–1.04)	0.112
Body Mass Index	26.1 ± 4	26.2 ± 4.2	0.99 (0.93–1.06)	0.838
Smoking status Never Former Currently	66/70 * 32 (45.7) 19 (27.1) 15 (21.4)	159/169 * 82 (51.6) 34 (20.1) 43 (25.4)	0.89 (0.51–1.56) 1.48 (0.77–2.83) 0.80 (0.41–1.56)	0.776 0.236 0.619
Family history of CAD	66/70 * 7 (10.6)	154/169 * 23 (13.6)	0.68 (0.28–1.66)	0.521
Diabetes	1 (1.4)	16 (9.5)	0.14 (0.02–1.07)	0.027
Hypertension	43 (61.4)	109 (64.5)	0.86 (0.49–1.53)	0.658
Hypercholesterolemia	15 (21.4)	44 (26)	0.77 (0.40–1.50)	0.511
Preoperative creatinine (μmol/L)	84 (69–98)	83 (72–97)	1.00 (0.99–1.01)	0.910
Previous MI	3 (4.3)	15 (8.9)	0.46 (0.13–1.64)	0.288
Previous PCI	2 (2.9)	9 (5.3)	0.52 (0.11–2.48)	0.516
Previous cardiac surgery	1 (1.4)	10 (5.9)	0.23 (0.03–1.84)	0.183

* Denominator represents number of patients for whom this information was known. Data are presented as *n* (%), mean ± SD or median (interquartile range). CAD = Coronary artery disease, MI = Myocardial infarction, PCI = Percutaneous coronary intervention, TAA = Thoracic aortic aneurysm.

**Table 2:** Perioperative characteristics.

	TAA	Non-TAA		
Surgery Type	*n* = 70	*n* = 169	OR (95% CI)	*p*-Value
Single AVR	1 (1.4)	34 (20.1)	0.06 (0.01–0.43)	<0.001
AVP	13 (18.6)	15 (8.9)	2.34 (1.05–5.22)	0.046
Concomitant CABG	11 (15.7)	35 (20.7)	0.71 (0.34–1.50)	0.471
Aortic procedures Root Ascending (Hemi)arch	58 (82.9) 65 (92.9) 14 (20)	56 (33.1) 18 (10.7) 3 (1.8)	9.75 (4.85–19.3) 109 (38–306) 13.51 (3.83–50)	<0.001 <0.001 <0.001
Other concomitant procedures				
Rhythm surgery	9 (12.9)	25 (14.8)	0.85 (0.38–1.93)	0.839
MVP	8 (11.4)	33 (19.5)	0.53 (0.23–1.22)	0.186
MVR	1 (1.4)	10 (5.9)	0.23 (0.03–1.84)	0.183
TVP	6 (8.6)	30 (17.8)	0.43 (0.17–1.10)	0.077

Data are presented as *n* (%), mean ± SD or median (interquartile range). AVP = Aortic valve plasty, AVR = Aortic valve replacement, CABG = Coronary artery bypass grafting, MVP = Mitral valve plasty, MVR = Mitral valve replacement, TAA = Thoracic aortic aneurysm, TVP = Tricuspid valve plasty.

**Table 3:** BAV and TAV patients vs. general population.

	**General Population**	**BAV**	**OR**	**95% CI**	***p*** **-Value**
Hypertension	16%	57.5%	7.1	4.49–11.21	<0.001
Hypercholesterolemia	9.1%	20.7%	2.61	1.49–4.57	0.002
Diabetes mellitus	6.6%	2.3%	0.33	0.08–1.38	0.162
CAD	3%	2.3%	0.76	0.18–3.24	1.000
	**General Population**	**TAV**	**OR**	**95% CI**	***p*** **-Value**
Hypertension	40.9%	67.1%	2.95	2.05–4.23	<0.001
Hypercholesterolemia	22.7%	27%	1.26	0.85–1.85	0.257
Diabetes mellitus	16.9%	9.9%	0.54	0.31–0.94	0.032
CAD	11.4%	10.5%	0.91	0.53–1.59	0.891

Data are presented as percentages. BAV = Bicuspid aortic valve, CAD = Coronary artery disease, CI = Confidence interval, OR = Odds ratio, TAV = Tricuspid aortic valve.

## Data Availability

Data are available upon reasonable request.

## Supplementary Materials

The following are available online at https://www.mdpi.com/article/10.3390/jcm12010272/s1, Figure S1: Sievers classification BAV patients, Figure S2: CAGE scores in BAV and TAV patients, Table S1: Study population vs. general population, Table S2: Baseline characteristics of BAV and TAV patients, Table S3: Perioperative characteristics of BAV and TAV patients.
