# Supplementary material for: Are Thoracic Aortic Aneurysm Patients at Increased Risk for Cardiovascular Diseases?
Source: J Clin Med. 2022 Dec 29;12(1):272. doi: 10.3390/jcm12010272 (PMC9821759; doi:10.3390/jcm12010272)
Supplement: Supplementary file 1 [file jcm-12-00272-s001.zip › jcm-2006935-supplementary.pdf]

Supplemental Table S1: Study population vs general population

	**General population**	**TAA**		**Non-TAA**	
Hypertension	32.2%	61.4%	p<0.001	64.5%	p<.001
Hypercholesterolemia	18.3%	21.4%	p=0.524	26%	p=.021
Diabetes mellitus	13.9%	1.4%	p=0.001	9.5%	p=.140
CAD	9.6%	4.3%	p=0.197	8.9%	p=.887

Data are presented as percentages. CAD = Coronary artery disease, TAA = Thoracic aortic aneurysm.

Supplemental Table S2: Baseline characteristics of BAV and TAV patients

	BAV	TAV		
Characteristic	n = 87	n = 152	OR (95% CI)	*P-value*
Male	72 (82.2)	103 (67.8)	2.28 (1.19-4.83)	.015
Age at surgery	54 (45-62)	67 (60-73)	1.09 (1.06-1.12)	< .001
Body Mass Index	26.1 ± 4.1	26.2 ± 4.1	1.01 (0.96-1.08)	.793
	82/87*	138/152*		
Family history of CAD	11 (12.6)	19 (12.5)	1.03 (0.46-2.29)	1.000
Diabetes	2 (2.3)	15 (9.9)	4.65 (1.04-20.86)	.035
Hypertension	50 (57.5)	102 (67.1)	1.54 (0.89-2.57)	.126
Hypercholesterolemia	18 (20.7)	41 (27)	1.43 (0.76-2.68)	.280
Preoperative creatinine (µmol/L)	83 (70-92)	83 (73-102)	1.01 (1.00-1.02)	.118
Previous MI	2 (2.3)	16 (10.5)	5.00 (1.12-22.29)	.021
Previous PCI	1 (1.1)	10 (6.6)	6.06 (0.76-48.14)	.060
Previous cardiac surgery	2 (2.3)	9 (5.9)	2.68 (0.57-12.67)	.336

*Denominator represents number of patients for whom this information was known. Data are presented as n (%), mean ± SD or median (interquartile range).

BAV = Bicuspid aortic valve, CAD = Coronary artery disease, MI = Myocardial infarction, PCI = Percutaneous coronary intervention, TAV = Tricuspid aortic valve.

Supplemental Table S3: Perioperative characteristics of BAV and TAV patients

	BAV	TAV		
Surgery type	n = 87	n = 152	OR (95% CI)	*P-value*
Single AVR	11 (12.6)	24 (15.8)	1.30 (0.60-2.79)	.572
AVP	8 (9.2)	20 (13.2)	1.49 (0.63-3.56)	.409
Concomitant CABG	13 (14.9)	33 (21.7)	1.58 (0.78-3.19)	.235
Aortic procedures				
Root	57 (65.5)	57 (37.5)	0.32 (0.18-0.55)	< .001
Ascending	37 (42.5)	46 (30.3)	0.59 (0.34-1.02)	.067
(Hemi)arch	9 (10.3)	8 (5.3)	0.48 (0.18-1.30)	.190
Other concomitant procedures				
Rhythm surgery	6 (6.9)	28 (18.4)	3.05 (1.21-7.69)	.020
MVP	5 (5.7)	36 (23.7)	5.09 (1.92-13.52)	< .001
MVR	1 (1.1)	10 (6.6)	6.06 (0.76-48.14)	.060
TVP	3 (3.4)	33 (21.7)	7.77 (2.31-26.16)	< .001

*Denominator represents number of patients for whom this information was known. Data are presented as n (%), mean ± SD or median (interquartile range).

AVP = Aortic valve plasty, AVR = Aortic valve replacement, BAV = Bicuspid aortic valve, CABG = Coronary artery bypass grafting, MVP = Mitral valve plasty, MVR = Mitral valve replacement, TAV= Tricuspid aortic valve, TVP = Tricuspid valve plasty

Supplemental Figure S1: Sievers classification BAV patients

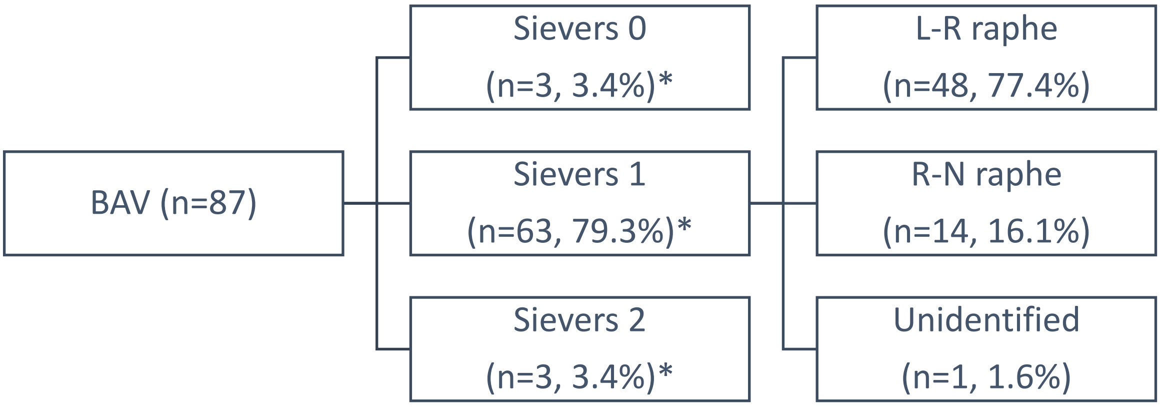

*Denominator represents number of patients for whom this information was known.

Supplemental Figure S2: CAGE scores in BAV and TAV patients

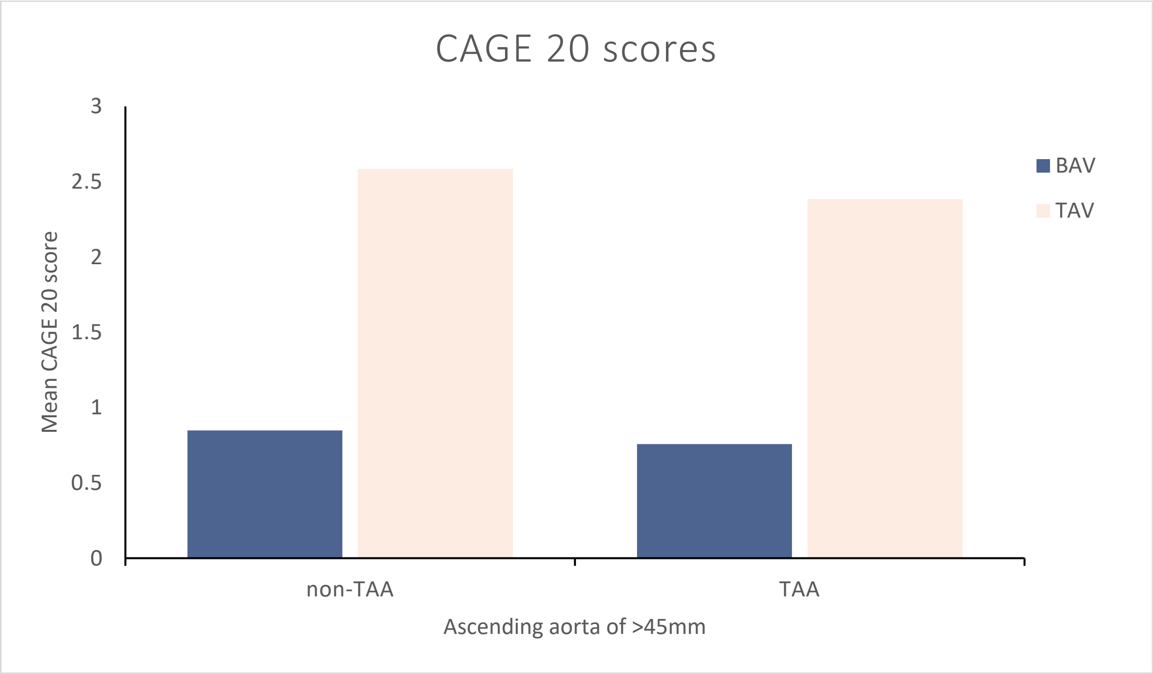

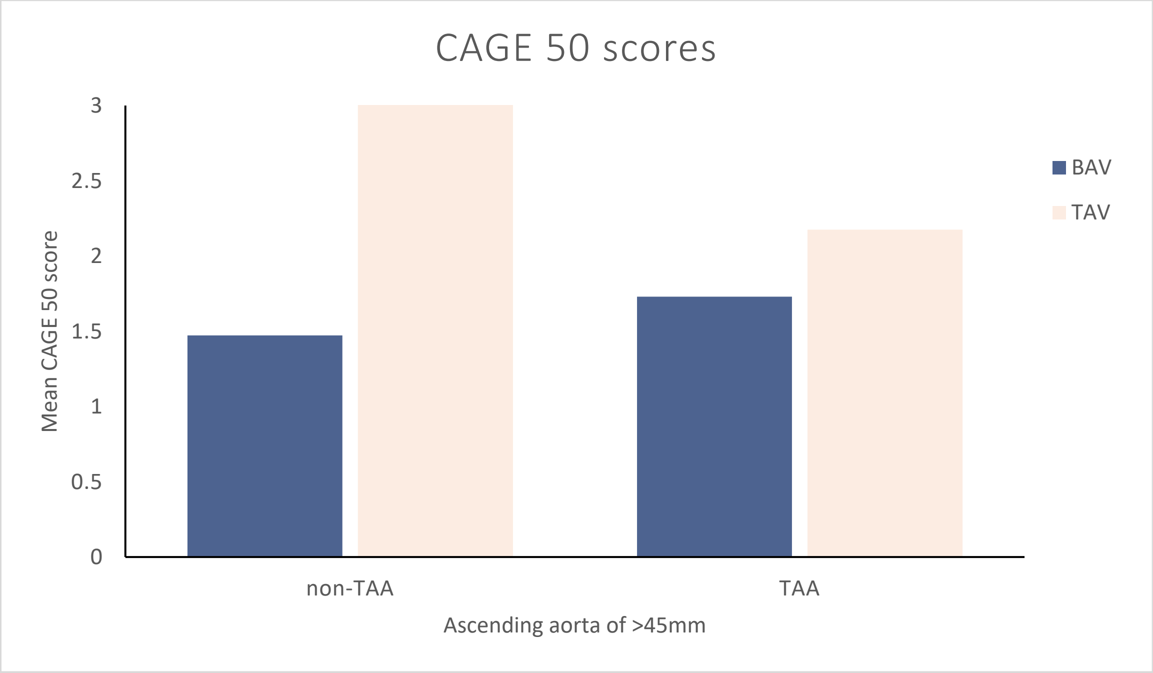

BAV = Bicuspid aortic valve, TAA = Thoracic aortic aneurysm, TAV = Tricuspid aortic valve.
